# On the rationale of population screening for chronic kidney disease: a public health perspective

**DOI:** 10.1186/s40985-015-0009-9

**Published:** 2015-11-05

**Authors:** Murielle Bochud

**Affiliations:** Chronic Disease Division, Institute of Social and Preventive Medicine, Lausanne University Hospital, Route de la Corniche 10, 1010 Lausanne, Switzerland

**Keywords:** Chronic kidney disease, Screening programme, Glomerular filtration rate, Urine analysis

## Abstract

Unlike opportunistic screening, population screening is accompanied by stringent quality control measures and careful programme monitoring. Sufficient evidence for benefit together with acceptable harms and costs to society are needed before launching a programme. A screening programme is a complex process organized at the population level involving multiple actors of the health care system that should ideally be supervised by public health authorities and evaluated by an independent and trustful body. Chronic kidney disease is defined by reduced glomerular filtration rate and/or presence of kidney damage for at least three months. Chronic kidney disease is divided into 5 stages with stages 1 to 3 being usually asymptomatic. Chronic kidney disease affects one in ten adults worldwide and its prevalence sharply increases with age. Kidney function is measured using serum creatinine-based, and/or cystatin C-based, equations. Markers of renal function show high intra-individual and inter-laboratory variabilities, highlighting the need for standardized procedures. There is also large inter-individual variability in age-related kidney function decline. Despite these limitations, chronic kidney disease, as currently defined, has been consistently associated with high cardiovascular morbidity and mortality and high risk of end-stage renal disease. Major modifiable risk factors for chronic kidney disease are diabetes, hypertension, obesity and cardiovascular disease. Several treatment options, ranging from antihypertensive and lipid-lowering treatments to dietary measures, reduce all-cause mortality and/or end-stage renal disease in patients with stages 1–3 chronic kidney disease. So far, no randomized controlled trial comparing outcomes with and without population screening for stages 1–3 chronic kidney disease has been published. Population screening for stages 1–3 chronic kidney disease is currently not recommended because of insufficient evidence for benefit. Given the current and future burden attributable to chronic kidney disease, randomized controlled trials exploring benefits and harms of population screening are clearly needed to prioritize resource allocations.

## Introduction

Chronic kidney disease (CKD) and its complications represent an enormous and increasing public health burden worldwide [1]. More than one in ten adults suffers from CKD in the general population [2], with a majority of people being in its early stages (i.e. 1 to 3) [2]. In the general population, the prevalence of CKD sharply increases with age [3]. CKD can be considered as a condition associated with premature ageing with accelerated vascular disease [4]. The large number of people with CKD, or at high risk of CKD (i.e. patients with hypertension, diabetes and/or CVD), implies that primary care providers and specialists other than nephrologists frequently encounter patients with CKD [5], a situation in which most CKD cases are diagnosed via opportunistic kidney function screening or automated eGFR reporting.

The aim of this review is to discuss the rationale and currently available evidence for, or against, population-based screening for CKD. The focus will be on the situation of screening asymptomatic individuals at early stages of CKD regardless of the presence or absence of CKD risk factors.

## Challenges in measuring renal function

Kidney function is usually measured by estimating glomerular filtration rate (GFR), which is currently considered to be the best index. A direct measurement of GFR is possible, such as by assessing urinary iothalamate or inulin clearance, but this is cumbersome and not suitable for route clinical or population screening. Several equations have been proposed to estimate GFR (eGFR) from serum creatinine and the currently recommended equation for adults is the Chronic Kidney Disease-Epidemiology Collaboration (CKD-EPI) equation [6]. The CKD-EPI equation also takes age, sex and race into account, because of their association with muscle mass, which influences the generation of creatinine. It is particularly challenging to accurately estimate eGFR in older adults, because the increase in serum creatinine reflecting reduced kidney function is paralleled by an age-related decrease in muscle mass [7]. Another issue is the need to calibrate serum creatinine assays across laboratories to use them to estimate GFR [8, 9]. Because creatinine depends on muscle mass and other factors, such as diet, that influence creatinine generation, there have been efforts to identify a marker of glomerular filtration that does not suffer from these limitations. Cystatin C, an endogenous protein produced by nearly all human cells that is freely filtered by the glomeruli, has recently been proposed as a new marker. Cystatin C-based equations to estimate GFR are now available [10–14]. Compared to creatinine, cystatin C-based equations better predicted all-cause mortality and cardiovascular events in people older than 65 years [15] as well as all-cause mortality and end-stage renal disease (ESRD) in general adult populations [11]. Cystatin C may be combined with creatinine to estimate GFR [11], as demonstrated by some recently published equations cited above [13, 14]. Markers of glomerular filtration (e.g. serum creatinine and cystatin C) and markers of kidney damage (e.g. albuminuria, renal biopsy findings) are also part of the tests used to define CKD-staging.

### How to diagnose chronic kidney disease?

CKD is defined by the Kidney Disease: Improving Global Outcomes Initiative (KDOQI) as abnormalities of kidney structure or function, present for more than 3 months, with implications for health [16]. The following criteria are considered as markers of kidney damage: albuminuria (albumin excretion rate of 30 mg/24 h or higher or albumin-creatinine ratio ≥ 30 mg/g); abnormal urine sediment; abnormal histology; structural abnormalities detected at imaging; history of kidney transplantation or present of kidney damage; eGFR < 60 mL/min/1.73 m^2^ for ≥3 months). KDIGO revised CKD staging by categorizing GFR into 6 stages and albuminuria into 3 stages (Table 1). In its early stages, CKD is almost always asymptomatic, which makes early CKD diagnosis of interest for population screening.

**Table 1:** Chronic kidney disease by GFR and albuminuria stages (adapted from [16] and [61])

			Albuminuria stages (mg/g)		
			A1: < 30	A2: 30–299	A3: >300
GFR stages (mL/min/1.73 m^2^)	G1	≥90	No CKD	CKD G1-A2	CKD G1-A2
	G2	60-89	No CKD	CKD G2-A2	CKD G2-A3
	G3a	45-59	CKD G3a-A1	CKD G3a-A2	CKD G3a-A3
	G3b	30-44	CKD G3b-A1	CKD G3b-A2	CKD G3b-A3
	G4	15-29	CKD G4a-A1	CKD G4a-A2	CKD G4a-A3
	G5	<15	CKD G5a-A1	CKD G5a-A2	CKD G5a-A3

*GFR* glomerular filtration rate, *CKD* chronic kidney disease

### Chronic kidney disease burden

CKD is associated with an elevated risk of all-cause mortality, the lower the kidney function, the higher the risk [17–19]. Albuminuria provides prognostic information with respect to all-cause and cardiovascular mortality as well as acute kidney injury, progression of CKD and ESRD, independently of estimated glomerular filtration rate (eGFR) [20, 21]. Compared to a person with an eGFR > 60 mL/min/1.73 m^2^, a person with an eGFR between 45 and 59 mL/min/1.73 m^2^ has 20 % higher mortality, a person with an eGFR between 30 to 44 mL/min/1.73 m^2^ has 80 % higher mortality, and a person with an eGFR below 30 has mL/min/1.73 m^2^ more than three-fold higher all-cause mortality [17]. The associated risks of cardiovascular events, and of hospitalizations, are of similar magnitudes than those observed for all-cause mortality at each of these eGFR categories, independently of diabetes, hypertension, dyslipidemia and prior cardiovascular disease (CVD) [17, 19]. The latest large-scale meta-analysis of cohorts highlighted a j-shaped relationship between eGFR and all-cause and cardiovascular mortality with the lowest mortality observed at an eGFR of about 95 mL/min/1.73 m^2^ [20]. The higher mortality observed in people with high eGFR may potentially reflect the deleterious effects of glomerular hyperfiltration or be the consequence of low creatinine generation in people with muscle wasting (e.g. cancer). In people with hypertension, diabetes or CVD, both low eGFR and the presence of albuminuria are independently associated with all-cause and cardiovascular mortality [22]. Also, there is no evidence that diabetes modifies the mortality risk associated with reduced eGFR or the presence of albuminuria [23].

## Natural history of renal function

The major risk factors for CKD include older age, diabetes, arterial hypertension, cardiovascular disease (CVD), obesity and selected ethnic groups [24–28]. Renal function gradually decreases with age [29, 30]. Yet, there is large inter-individual variability in age-related kidney function decline [7, 28, 31–33], with some people being rapid progressors and others non-progressors. So far, our ability to predict rapid progression has been limited [34]. In longitudinal studies, the average rate of eGFR decline varied from 0.4 to 1.2 mL/min/1.73 m^2^/year in healthy adults, was usually higher in patients with comorbidities or in older people (i.e. 1 to 2 mL/min/1.73 m^2^/year) and even higher in patients with CKD (i.e. 2 to 5 mL/min/1.73 m^2^/year) [30]. People with rapid age-related eGFR decline have higher all-cause and cardiovascular mortality, independently of baseline eGFR level [7, 28, 31, 35], which underscores the importance of taking not only point-estimates of kidney function into account, but also longitudinal variation. Rapid eGFR decline was also associated with higher incidence of cardiovascular events [33]. There is however some uncertainty regarding how to best define rapid eGFR decline [30]. Some studies have used absolute [7, 32, 33] and others relative (i.e. percent changes) [16, 31, 35] eGFR differences. Of interest, people with CKD stage 3, in whom an increase in eGFR during follow-up is observed, tend to have higher mortality than those with a stable eGFR with time [31].

Large scale studies found obesity to represent a risk factor for the development of CKD and ESRD, independently of classical CKD risk factors [36–39], although this independence is not clear for stage 3 CKD [40]. Obesity also is a risk factor for CKD in non-diabetic people [41]. The mechanisms by which obesity could negatively influence renal function are currently unclear [42]. Obesity raises the important issue of whether or not to index kidney function for body surface area, as is currently the case [43, 44]. Such indexing may lead to underestimate eGFR or, by contrast, may mask obesity-related glomerular hyperfiltration [43, 44], a potential marker of future kidney function deterioration.

Although little prospective population-based data are available [30], there is also substantial intra-individual variability in kidney function trajectory over time, with some people displaying a linear decline and others a non-linear decline (e.g. eGFR increases and then rapidly decreases), such as observed in patients with diabetes and/or obesity. The dynamic variability in eGFR has been inconsistently associated with higher risk of ESRD [45, 46] and more research is needed in this area, in particular to explore the effect, and pertinence, of indexing GFR for body surface area.

## Principles of population-based screening

The management of screening is a public health service that needs (1) to identify programmes that do more harm than good at affordable costs and (2) to ensure that the quality of the programmes are continuously monitored [47].

### Elements of a screening programme

“Screening is a programme not a test” [47]. « One of the aims of screening is to control a disease at the population level ». According to Gray [47], a screening programme is composed of five basic elements: (1) a target population to be invited at specified intervals; (2) one or more screening tests; (3) one or more diagnostic tests; (4) treatment options and (5) quality management. A screening programme often has to deal with opportunistic screening, i.e. screening tests that are made outside of the programme, that are often conducted by the private health care sector and are usually not subject to the same quality controls and evaluation procedures. Depending on the country and the organization of the health care system, opportunistic screening may create different problems and consequences.

In 1968, Wilson and Jungner published their seminal report entitled “Principles and practice of screening for disease” [48]. The idea was to guide the selection of conditions that would be suitable for screening. Among other criteria, the condition needs to be detectable at an early stage and be treatable. CKD certainly fulfils these two criteria, in that kidney function can be easily measured via blood and urine testing (even if one has to acknowledge the difficulty to precisely and accurately assess GFR in selected population such as obese, elderly, frail, cachectic or cirrhotic people) and drugs are available to slow down the decrease in renal function in selected groups. The criteria proposed by Wilson and Jungner are listed in Table 2, in which a comment was added to relate them to the specific situation of CKD screening. Additional criteria have since been added, such as those proposed by the UK National Screening Committee (Table 3). These new criteria aim to put more emphasis on potential harms of screening programmes, to clarify the level of evidence needed to consider a programme as efficient (i.e. high quality randomized controlled trials) and to underline the importance of quality assurance and programme evaluation [49]. Andermann et al. [50] recently published revised screening criteria (Table 4).

**Table 2:** Wilson & Jungner screening criteria in the context of CKD screening (adapted from [48])

	Criteria	Comment regarding CKD screening
1	The condition is an important health problem.	CKD affects one in 10 adults worldwide. CKD increases the risk of all-cause and CV mortality and ESRD.
2	There should be an accepted treatment for patients with recognized disease	Treatment would need to be adapted to the presence of risk factors and co-morbidities (e.g. hypertension, diabetes, CVD, etc.)
3	Facilities for diagnosis and treatment should be available.	Diagnosis and treatment are routinely available in hospitals and health care centers.
4	There should be a recognizable latent or early symptomatic stage.	CKD in its early stages (1–3) is almost always asymptomatic.
5	There should be a suitable test or examination.	Serum creatinine, serum cystatin C and urinary microalbumin represent suitable tests to detect CKD.
6	The test should be acceptable to the population.	Serum creatinine, serum cystatin C and urinary microalbumin are non-invasive and affordable tests.
7	The natural history of the condition, including development from latent to declared disease, should be adequately understood.	Several cohort studies have shown a linear age-related decrease in renal function, but there are large inter-individual differences. People affected with CKD either die from CVD or develop ESRD (dialysis or kidney transplantation).
8	There should be an agreed policy on whom to treat as patients.	There is high-quality evidence to recommend treatment with angiotensin II-receptor blockers in patients with CKD stages 1 to 3 [24], although evidence is lower in non-diabetic patients [58].
9	The cost of case-finding (including diagnosis and treatment of patients diagnosed) should be economically balanced in relation to possible expenditure on medical care as a whole.	This would need to be determined within each health care system.
10	Case-finding should be a continuing process and not a “once and for all” project.	Regular assessments of renal function would be quite easy to put in place.

*CKD* chronic kidney disease, *CVD* cardiovascu lar disease, *ESRD* end-stage renal disease

**Table 3:** Criteria for appraising the viability, effectiveness and appropriateness of a screening programme — 2003 (UK National Screening Committee) (authorization obtained from publisher)

1. The condition should be an important health problem.
2. The epidemiology and natural history of the condition, including development from latent to declared disease, should be adequately understood and there should be a detectable risk factor, disease marker, latent period or early symptomatic stage.
3. All the cost-effective primary prevention interventions should have been implemented as far as practicable.
4. If the carriers of a mutation are identified as a result of screening the natural history of people with this status should be understood, including the psychological implications.
5. There should be a simple, safe, precise and validated screening test.
6. The distribution of test values in the target population should be known and a suitable cut-off level defined and agreed.
7. The test should be acceptable to the population.
8. There should be an agreed policy on the further diagnostic investigation of individuals with a positive test result and on the choices available to those individuals.
9. If the test is for mutations the criteria used to select the subset of mutations to be covered by screening, if all possible mutations are not being tested for, should be clearly set out.
10. There should be an effective treatment or intervention for patients identified through early detection, with evidence of early treatment leading to better outcomes than late treatment.
11. There should be agreed evidence-based policies covering which individuals should be offered treatment and the appropriate treatment to be offered.
12. Clinical management of the condition and patient outcomes should be optimised in all healthcare providers prior to participation in a screening programme.
13. There should be evidence from high-quality randomised controlled trials that the screening programme is effective in reducing mortality or morbidity. Where screening is aimed solely at providing information to allow the person being screened to make an ‘informed choice’ (for example, Down’s syndrome and cystic fibrosis carrier screening), there must be evidence from high-quality trials that the test accurately measures risk. The information that is provided about the test and its outcome must be of value and readily understood by the individual being screened.
14. There should be evidence that the complete screening programme (test, diagnostic procedures, treatment/intervention) is clinically, socially, and ethically acceptable to health professionals and the public.
15. The benefit from the screening programme should outweigh the physical and psychological harm (caused by the test, diagnostic procedures and treatment).
16. The opportunity cost of the screening programme (including testing, diagnosis and treatment, administration, training and quality assurance) should be economically balanced in relation to expenditure on medical care as a whole (i.e. value for money).
17. There should be a plan for managing and monitoring the screening programme and an agreed set of quality assurance standards.
18. Adequate staffing and facilities for testing, diagnosis, treatment, and programme management should be available prior to the commencement of the screening programme.
19. All other options for managing the condition should have been considered (for example, improving treatment and providing other services), to ensure that no more cost-effective intervention could be introduced or current interventions increased within the resources available.
20. Evidence-based information, explaining the consequences of testing, investigation, and treatment, should be made available to potential participants to assist them in making an informed choice.
21. Public pressure for widening the eligibility criteria for reducing the screening interval, and for increasing the sensitivity of the testing process, should be anticipated. Decisions about these parameters should be scientifically justifiable to the public.
22. If screening is for a mutation, the programme should be acceptable to people identified as carriers and to other family members.

**Table 4:** Emerging screening criteria proposed over the past 40 years in the context of CKD screening (adapted from Andermann et al. [50])

	Criteria	Comment regarding CKD screening
1	The screening programme should respond to a recognized need.	The current situation of population ageing with increasing incidence of CKD and ESRD and their associated costs strongly suggest that there is a need.
2	The objectives of screening should be defined at the outset.	The objective should be to decrease all-cause and CV mortality as well as ESRD incidence and mortality and also to improve the quality of life of people living with CKD.
3	There should be a defined target population.	Although no consensus exists for CKD screening, the sharp age-related increase in CKD prevalence suggests starting screening after the age of 50 years.
4	There should be scientific evidence of screening programme effectiveness.	No such evidence currently exists.
5	The programme should integrate education, testing, clinical services and programme management.	No programme is currently being tested.
6	There should be quality assurance, with mechanisms to minimize potential risks of screening.	No programme is currently being tested.
7	The programme should ensure informed choice, confidentiality and respect for autonomy.	No programme is currently being tested.
8	The programme should promote equity and access to screening for the entire target population.	No programme is currently being tested.
9	Programme evaluation should be planned from the outset.	No programme is currently being tested.
10	The overall benefits of screening should outweigh the harm.	No such evidence currently exists.

*CKD* chronic kidney disease, *CVD* cardiovascular disease, *ESRD* end-stage renal disease

### What could be used as a screening test for chronic kidney disease?

A blood test for serum creatinine and a spot urine for albumin-to-creatinine ratio could represent the first screening tests for CKD. For those testing positive, a confirmation, i.e. a diagnostic test, should then be done by reassessing eGFR, potentially using both serum creatinine and cystatin C, and urine analysis at three months, and likely adding a non-invasive imaging of the kidneys. Cost considerations and analytic validity of the tests need to be carefully considered when deciding on which tests to use within a given context. In particular, the measurement of cystatin C currently has a much higher cost (about 10 times higher) than that of creatinine.

### Population-based versus opportunistic screening

Population-based screening programmes target groups of people and not individuals. As such, they can be contrasted with opportunistic screening that individuals seek from their medical doctor, outside of a formal population screening programme. When making the decision to start or not a screening programme, policy makers always consider cost issues, the idea being to maximize the value obtainable from the available resources [51]. Health authorities have to consider not only the evidence and needs of the population but also the values of that population [51]. It may therefore well be that a specific programme is adapted to the values of one population but not to those of another population, even if the programme is evidence-based. Economists, epidemiologists, and public health professionals gather evidence about a programme, yet values have to be decided by the public, or usually, by their elected representatives [51].

## CKD screening recommendations

Current guidelines of the American College of Physicians [24] recommend against screening for CKD in asymptomatic adults without risk factors for CKD. There is only low-quality evidence available so that this recommendation is of weak grade. The UK National Screening Committee does not recommend population screening for kidney disease in general (http://legacy.screening.nhs.uk/kidneydisease). The US Preventive Services Task Force (USPSTF, www.uspreventiveservicestaskforce.org) considers that there is insufficient evidence to assess the benefits and harms of screening for CKD in asymptomatic individuals in the absence of diabetes or hypertension. No population-based study explored the sensitivity and specificity of screening for CKD using eGFR or albuminuria or both, neither once nor multiple times [24, 52]. So far there are no controlled clinical trials comparing outcomes with and without CKD screening in the population [52].

## What criteria should be met for population-based screening to be recommended?

In the absence of evidence, such as is currently the case, population-based screening for CKD cannot be recommended [53]. Given the high burden associated with CKD and its complications, it would seem appropriate to initiate a randomized controlled trial for CKD screening in the general adult population. The project should explore which screening modality would carry the optimal sensitivity and specificity in the context of CKD screening: creatinine-based eGFR alone? albumin-to-creatinine ratio in spot urine? both tests? single versus multiple tests? Furthermore the best age range to define the target population would need to be determined. Considering the age-dependent prevalence, it is likely that CKD screening would not be very efficient before the age of 50 years. The optimal screening interval should also be explored (yearly? every 2 years? etc.). The current KDIGO definition does not factor age into the CKD staging system, which has been repeatedly criticized [54] and debated [55]. A detailed discussion about the need for revised criteria for CKD-staging is beyond the scope of this review, but this clearly represents an important question when choosing a proper CKD screening test.

There is good evidence that early lifestyle and pharmacologic interventions can slow down CKD progression and reduce CV risk [56]. There is no uniform treatment option for patients with CKD as the treatment strategy strongly depends on the presence of comorbidities, such as diabetes, dyslipidemia, hypertension and on overall cardiovascular risk [21]. In diabetic patients, ACE inhibitor treatment reduces the doubling of serum creatinine level by 42 % (OR[95 % CI]: 0.58 [0.32–0.90]) [57]. There is a lack of trials evaluating CKD treatment in non-diabetic and non-hypertensive individuals. Blood pressure should be adequately controlled in patients with CKD, with tighter control in the presence of albuminuria [21]. There is high-quality evidence to recommend treatment with angiotensin II-receptor blockers in patients with CKD stages 1 to 3 [24], although evidence is lower in non-diabetic patients [58]. Diabetes, if present, should be adequately controlled [21]. Current KDIGO guidelines recommend lipid lowering treatment in patients with stage 1–5 CKD aged older than 50 years [59]. For CKD patients younger than 50 years, lipid lowering treatment is recommended in case of diabetes, known vascular disease or whenever CV risk is higher than 10 % [59]. Lifestyle modifications expected to slow down CKD progression include low dietary salt and protein intake, regular physical exercise, weight maintenance and smoking cessation [21]. Another important issue in patients with CKD is that of adapting dosage for drugs cleared by the kidney. Detailed guidelines regarding drug dosage adjustment in patients with CKD have been published [60].

### Potential harms of screening

As highlighted by Gray [47], screening programmes generate specific harms that differ from those encountered in clinical care. In usual clinical care, a person seeks the help of a professional because of a specific problem. After having received adequate information, this person accepts the risk that the treatment might involve. In a screening programme, some people (false positive screening tests) may suffer from adverse effects of screening (e.g. colon perforation following a colonoscopy) without having the condition (e.g. colon cancer) for which they have been screened [47]. It is therefore of paramount importance that screening programmes gather evidence about the harm they may generate [47]. For the situation of CKD screening, the screening and diagnostic tests (blood and urine sampling) do not generate life-threatening complications, although the psychological harm of being labeled as suffering from CKD cannot be excluded. Screening and monitoring harms are poorly described for CKD so far [52].

## Conclusions

CKD in its early stages is asymptomatic. CKD affects one in ten adult in the general population and its prevalence sharply increases with age. CKD is associated with high cardiovascular morbidity and mortality and high risk of ESRD. In the absence of evidence for benefit, most public health authorities currently recommend against population-based screening for CKD. Considering the weakness of the available evidence, the increasing public health burden of CKD and the current context of population ageing, it seems appropriate and timely to initiate randomized clinical trials comparing outcomes with and without CKD screening in the general adult population. Such trials are needed to explore whether benefits, if any, outweigh harms. If net benefits are demonstrated at the population level, cost issues need to be explored but these considerations will strongly depend on the resources available in any specific country or region.

## Abbreviations

CKD: Chronic kidney disease
ESRD: End-stage renal disease
eGFR: Estimated glomerular filtration rate
CKD-EPI: Chronic kidney disease-epidemiology collaboration equation
KDOQI: Kidney disease: improving global outcomes initiative
CVD: Cardiovascular disease
